# A small-fish model for behavioral-toxicological screening of new antimalarial drugs: a comparison between erythro- and threo-mefloquine

**DOI:** 10.1186/s13104-015-1088-x

**Published:** 2015-04-02

**Authors:** Hans Maaswinkel, Liqun Zhu, Wei Weng

**Affiliations:** Research and Development, xyZfish, 2200 Smithtown Ave, Ronkonkoma, NY 11779-7329 USA

**Keywords:** Erythro-mefloquine, Threo-mefloquine, Behavioral toxicology, Goldfish, Zebrafish, Antimalarial drugs, 3D tracking

## Abstract

**Background:**

New antimalarial drugs need to be developed because over time resistance against the existing drugs develops. Furthermore, some of the drugs have severe side effects. Here we describe a behavioral small-fish model for early detection of neurotoxic effects of new drugs. As case example we compare the effects of two mefloquine diastereomers on the behavior of goldfish using an automated 3D tracking system.

**Findings:**

In a preliminary experiment, the overall toxic effects in terms of motor and respiratory impairments were determined during a 3-hour exposure to the drugs at relatively high doses (21.5 and 43 mgL). In the second experiment, behavioral testing was performed 24 h after a 3.5-h drug exposure to a low dose (14.25 mgL) of either drug. For the two high doses, erythro-mefloquine resulted in severe motor problems and respiratory problems occurred. In goldfish treated with threo-mefloquine, at 43 mgL the motor/respiratory impairments were less severe and at 21.5 mgL no such problems were observed. For the lower dose (14.25 mgL), erythro-mefloquine reduced locomotion. There was also a tendency for increased freezing, and the preference for quadrant two of the observation container was increased. No behavioral effects of threo-mefloquine were found.

**Conclusions:**

The results demonstrate that in goldfish exposed to the drugs dissolved in the water, threo-mefloquine has less severe toxic effects as compared to erythro-mefloquine. These findings are consistent with other studies and support the usefulness of the small-fish model for predicting adverse effects of new antimalarial drugs during the initial phases of drug development.

## Background

In 2012, 207 million incidences of malaria have been reported causing the death of an estimated 627 thousand people [1]. *Plasmodium falciparum* accounts for approximately 75% of the malaria cases and is the most fatal variant, at least in Africa. Currently, mefloquine, doxycycline, a combination of atovaquone and proguanile (Malarone), and chloroquine (for areas where resistance is not yet prevalent) are the drugs used for prophylaxis of malaria caused by *P. falciparum*. Mefloquine (or more precisely, erythro-mefloquine) was traditionally preferred for use by the US military because it has to be administered on a weekly basis. It is still perceived as a standard, with tafenoquine as one of the few alternatives [2]. Most other drugs have to be administered daily, which increases the risk of reduced compliance. However, mefloquine has more severe and longer-lasting side effects than the other antimalarial drugs. Therefore the US military decided to replace it with doxycycline (when not contraindicated) [3].

The most severe side effects of mefloquine, especially when administered over a prolonged period (as is necessary for prophylactic use), include increased anxiety, depression, sleep disturbances, nightmares, hallucinations and, in some cases, psychotic attacks or convulsions [4-7]. These effects may last for months or even years after the last drug intake. Acute adverse effects become more obvious when mefloquine is used for treatment of the disease, in which case a 5 times higher dose (1250 mg) is administered [8,9]. In a rat model, neurological effects such as impaired balance have been described [10]. Motor learning was also impaired in humans [11]. In healthy adults, administration of 1250 mg mefloquine (i.e. therapeutic dose) resulted in vertigo in 96% of the participants. Other common symptoms included nausea, insomnia and depression. The symptoms lasted up to 3 weeks after drug administration [12].

Because mefloquine is the prophylactic drugs with the longest half-life time, much effort has been put into understanding the toxic mechanism of mefloquine [10,13-16] and into developing analogues that combine its advantages with diminished adverse effects [17-19]. Another method to increase the therapeutic window of mefloquine might be isomer selection. Mefloquine is composed of four isomers. These are divided into two groups (called diastereomers) each consisting of two enantiomers (namely a [+]- and a [−]-enatiomer), which are each other’s mirror images within the groups but not across groups. The first group is called threo-mefloquine (WR-177,602) defined by the R,R and S,S configurations (as group: R*,R*). The second group is called erythro-mefloquine (WR-142,490) defined by the R,S and S,R configurations (as group: R*,S*). Erythro-mefloquine as racemic mixture is the drug clinically used against malaria. In this study we compare the effects of acutely administered (racemic) erythro-mefloquine and (racemic) threo-mefloquine in goldfish on behavioral side-effects (as determined 24 h after exposure). The overall purpose of this study is to assess how a small-fish model can be applied to determine behavioral-toxic effects of new antimalarial candidates in the early stages of drug development.

## Findings

### Animals

Juvenile goldfish (*Carassius auratus*) were purchased at a local pet-store (Country Critters, Patchogue, New York), with an average body-length of approx. 60 mm at the time of testing. They were maintained in 30-gallon aquariums at room temperature (approx. 23°C). Light regimen: 14 h lights on (6:00 – 20:00), 10 h lights off. The goldfish were fed three times a day with TetraMin goldfish flakes. Occasionally, we fed them live brine shrimp larvae. Before starting the experiments, the goldfish were habituated to the laboratory conditions for at least three weeks. The health of the goldfish was daily assessed by visual inspection of their appearances. In total, 366 goldfish were used. After the experiments, the goldfish were euthanized with 300 mgL tricaine methanesulfate (MS-222). All animal experiments were approved by the Stony Brook University Institutional Animal Care and Use Committee (IACUC # 10–1715, incl. amendment).

### Drugs

Erythro-mefloquine and threo-mefloquine were purchased from Bioblocks Inc. (product numbers: QUO24-1 and QUO25-1, respectively). Bioblocks Inc. assured us that QU025-1 is indeed a racemic mixture, in contrast to what has been reported in [20]. The solutions were freshly prepared every day. The assignment of the goldfish to experimental groups was randomized. The experimenter was not aware of the group assignments.

### Behavioral testing apparatus

**Figure 1:**
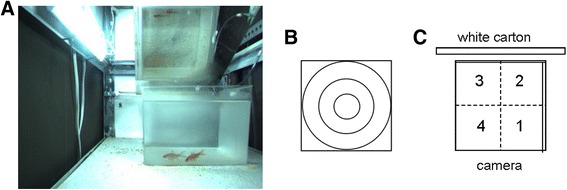
**Behavioral recording apparatus. (A)** The observation container as seen on the recording. Two juvenile goldfish swim close to the front wall. The top view can be seen on the overhead mirror. The combination of both views is necessary to calculate the 3D trajectories of the fish. Note that the container walls to the right and the back are semi-transparent (to reduce mirroring) and the other two walls are fully transparent. **(B)** Schematic presentation of the radial zones: the center, middle and outer zones have an outer diameter of 1/3, 2/3 and 3/3 of the width of the container. The corner zone is the combination of the areas of the four corners outside the outer zone. **(C)** Schematic presentation of the locations of the four quadrants. Note that they have distinct characteristics in terms of walls (transparent or semi-transparent; the latter are presented by double lines) and in terms of the surrounding physical features (e.g. white carton on the back, green curtain on all other sides, different distances from curtain, and presence of the camera at one side).

Twelve recording apparatus (Figure 1; further described in [21,22]) were used with each consisting of an compartment (length, 91 cm; width, 46 cm; height, 56 cm) and enclosed by a dark green curtain. The observation container had two transparent and two semi-transparent walls, length/width of approx. 25 cm, (with the two semi-transparent wall very slightly tapering off toward the bottom) and height of 18 cm (water level: 13.5 cm). The container was placed close to one side on the long axis of the observation compartment and a camera (Bumblebee 2; Point Grey research Inc, Vancouver, Canada) was placed close to the other side. A white carton was placed behind the container to increase visual contrast for better recording. Above the container a mirror was suspended at an angle such that the top and front views could be recorded simultaneously. LED bars above the observation container provided the necessary illumination (approx. 3160 K) of about 800 lux at water level. Images were recorded at approx. 40 frames per second. After recording, the centroid of every goldfish was determined by the software and the trajectories were reconstructed. These were then used for further analysis to extract travel distance in all three dimensions, duration freezing, freeze and burst frequency, and spatial distribution parameters (for Behavioral analysis, see below). The software corrected for the considerable error introduced by light refraction and for mirror distortion. Furthermore, (e.g. jigging) noise was reduced by software filters.

### Preliminary experiment: overall behavioral effects at 21.5 and 43 mgL

In experiment 1, (at approx. 13:00) goldfish were exposed for three hours to either 43 or 21.5 mgL erythro-mefloquine, threo-mefloquine or water (n = 22 per group for the higher dose and n = 20 per group for the lower doses). The maximal therapeutic dose in humans is approx. 1250 mg, which equals about 18 mgL. To account for reduced uptake in goldfish (which is unknown in this study) we chose a maximal dose of 43 mgL. The gross-behavioral changes as could be observed by the naked eye were noted, though not systematic classified, since the goal of this preliminary experiment was to determine whether this dose was adequate for behavioral testing.

Especially breathing pattern and motor coordination/balance of the goldfish, were visually monitored. Mild to moderate respiratory or motor problems were not alleviated with analgesics/anesthetics in order not to interfere with the purpose of the experiment. However, when a goldfish was severely impaired, usually lying on its side at the bottom of the tank, often accompanied by heavy breathing and sometimes short bursts of uncoordinated bursts of frantic swim bouts, we decided to euthanize the fish with 300 mgL tricaine methanesulfate (MS-222). Note that in this case, those fish were counted as ‘not survived’, although not many fish actually died before euthanasia was performed.

### Behavioral testing at 14.5 mgL

On the day preceding the days of recordings (at approx. 13:00), twenty-four goldfish were exposed to the 4-L drug-solutions (8 fish per container for any of the three conditions: water, 14.25-mgL erythro-mefloquine or 14.25-mgL threo-mefloquine) in containers similar to the observation containers. After 3.5 h the fish were placed in clean conditioned water (in similar containers; aeration was provided). Approximately 24 h later, the behavioral tests were performed. Immediately after placing pairs of goldfish in the observation containers, the recordings were started. Goldfish were tested in pairs, because single juvenile goldfish in a novel environment freeze most of the time. Thus four pairs per experimental group were tested on any of the ten experimental days. The distribution of goldfish pairs over the groups was: controls, n = 39; threo-mefloquine, n = 39; erythro-mefloquine, n = 36 (originally per group, n = 40; however, technical problems resulted in excluding some of the recordings). Twelve observation chambers were used. The distribution of the three experimental groups over the chambers was balanced using a rotating pattern as to avoid biasing the experiment.

The following behavioral endpoints were analyzed: distance travelled (3D, horizontal and vertical components), duration and frequency freezing (speed threshold: 2 mm/s; duration threshold: 1 s) and burst frequency (acceleration threshold: 2 m/s2), vertical distribution (average distance from bottom and time spent in any of the three equal depth layers: bottom, middle, top), and horizontal distribution (average distance from center, time spent in any of the four radial zones, and time spent in any of the four quadrants). For locations of radial zones and quadrants, see Figure 1. All observation data were obtained by averaging the data for the pairs.

Because the normality assumption as tested by Shapiro-Wilk tests and the equality-of-variances assumption as tested by Levene’s tests were in many cases not met, we decided to use Kruskal-Wallis tests in all cases to determine whether any significant behavioral effects were present. If so, we applied post-hoc Dwass-Steel-Critchlow-Fligner tests for pair-wise comparisons. Since we tested four groups of variables (travel distances as kinematic variables, freezing and bursts as dynamic variables, vertical distributions and horizontal distributions), we chose α = 0.05/04 = 0.0125 for the Kruskal-Wallis H-tests. For the post-hoc pair-wise comparisons, we applied Bonferroni’s corrections, i.e. α = 0.0125/3 = 0.0041. Because of the very rigorous statistical criteria, we also reported ‘tendencies’ (for p < 0.05), however, without drawing definitive conclusions.

### Data availability

The data sets supporting the results of this article are included within the article.

### Results of preliminary experiment: overall behavioral effects at 21.5 and 43 mgL

Against our original expectations, many goldfish died at a dose just twice the therapeutic dose in humans. Note, as explained above, that fish that showed signs of severe motor/respiratory impairments were euthanized out of humane considerations and were counted as having ‘died’. This limits somewhat the stringency of this part of the study.

After being exposed for 3 h to 43 mgL erythro-mefloquine, all 22 goldfish had perished (i.e. 0% survival). In the group exposed to 43 mgL threo-mefloquine, only one goldfish had died after 3 h (i.e. 95.5% survival); however, most goldfish showed at least some minor signs of motor problems.

After being exposed to 21.5 mgL erythro-mefloquine for 3 h, 7 out of 20 goldfish had died (i.e. 65% survival). In contrast, all goldfish exposed to 21.5-mgL threo-mefloquine or to water survived throughout the experiment and showed no obvious abnormal motor or respiratory responses.

### Results of behavioral testing at 14.5 mgL

**Figure 2:**
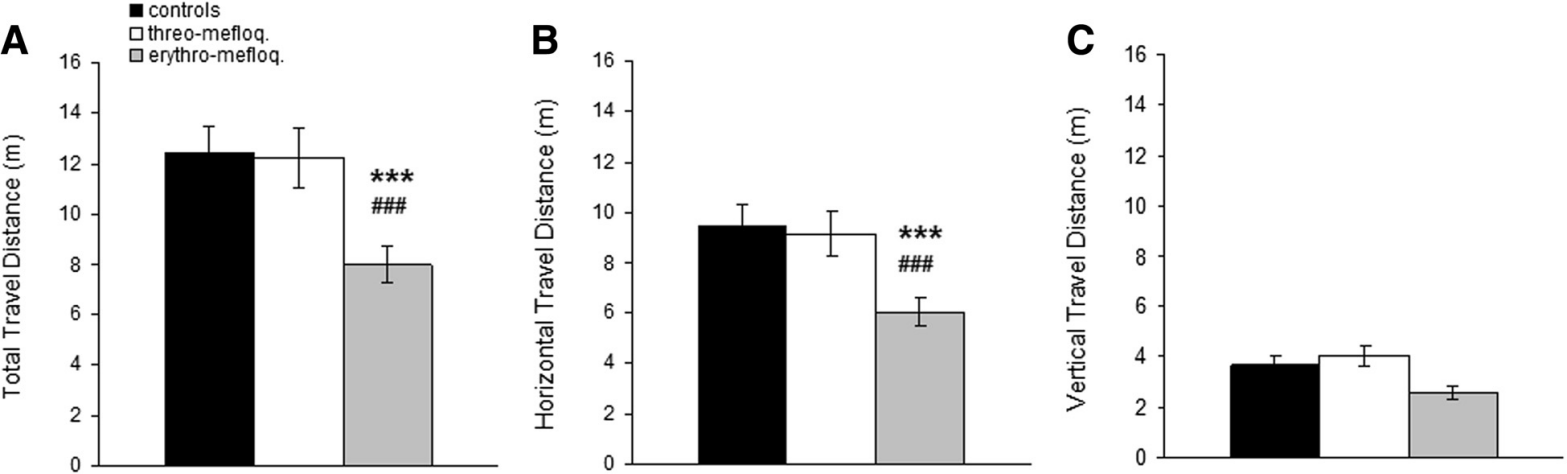
**Travel distance. (A)** Total or 3D travel distance. **(B)** Horizontal travel distance. **(C)** Vertical travel distance. Mean ± SEM are presented. Comparison between erythro-mefloquine and water: ***, p < 0.0005. Comparison between erythro-mefloquine and threo-mefloquine: ###, p < 0.0005.

Kruskal-Wallis test revealed that there was a significant effect (H = 10.37, p < 0.01) of treatment on 3D travel distance (Figure 2A). The erythro-mefloquine group travelled significantly less than the threo-mefloquine group (p < 0.0005) and significantly less than the control group (p < 0.0005). On the other hand, there was not significant difference between the threo-mefloquine and the control groups. Similar differences were observed for horizontal travel distance (Figure 2B): overall there was a significant treatment effect (H = 10.04, p < 0.01). The erythro-mefloquine group travelled significantly less in the horizontal plane than the threo-mefloquine group (p < 0.0005) and the control group (p < 0.0005), but the latter two groups were not significantly different from each other. On the other hand, no significant differences were observed in regard to vertical travel distance (Figure 2C). Only a tendency to such a difference was observed (p = 0.0133). In fact, all fish stayed relatively close to the bottom of the tank (see below), thus vertical traveling was minimal in all cases.

**Figure 3:**
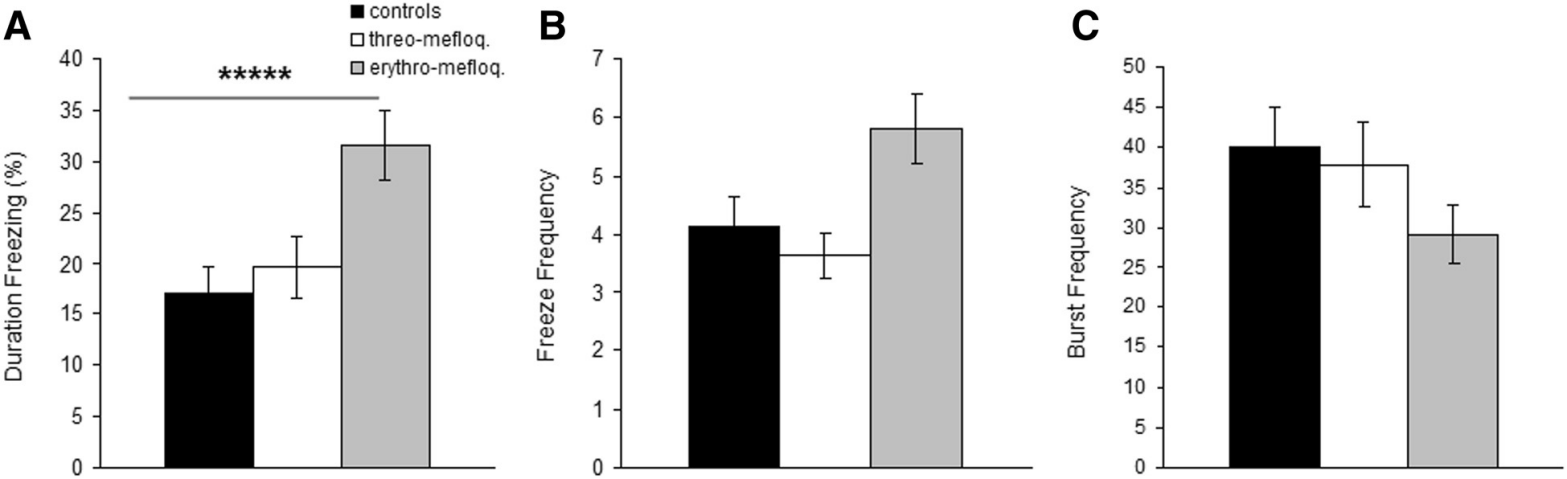
**Freezing and bursts. (A)** Duration freezing. **(B)** Freeze frequency. **(C)** Burst frequency. Mean ± SEM are presented. Overall effect of treatment: *****, p < 0.0001.

Although for duration freezing (Figure 3A) there was a significant treatment effect (H = 13.92, p < 0.001), Dwass-Steel-Fligner post-hoc test did not reveal any significant differences between pairs of treatments. For freeze frequency (Figure 3B) and burst frequency (Figure 3C) no statistical differences were observed, although for the former a tendency (p = 0.018) to such a difference was observed.

**Figure 4:**
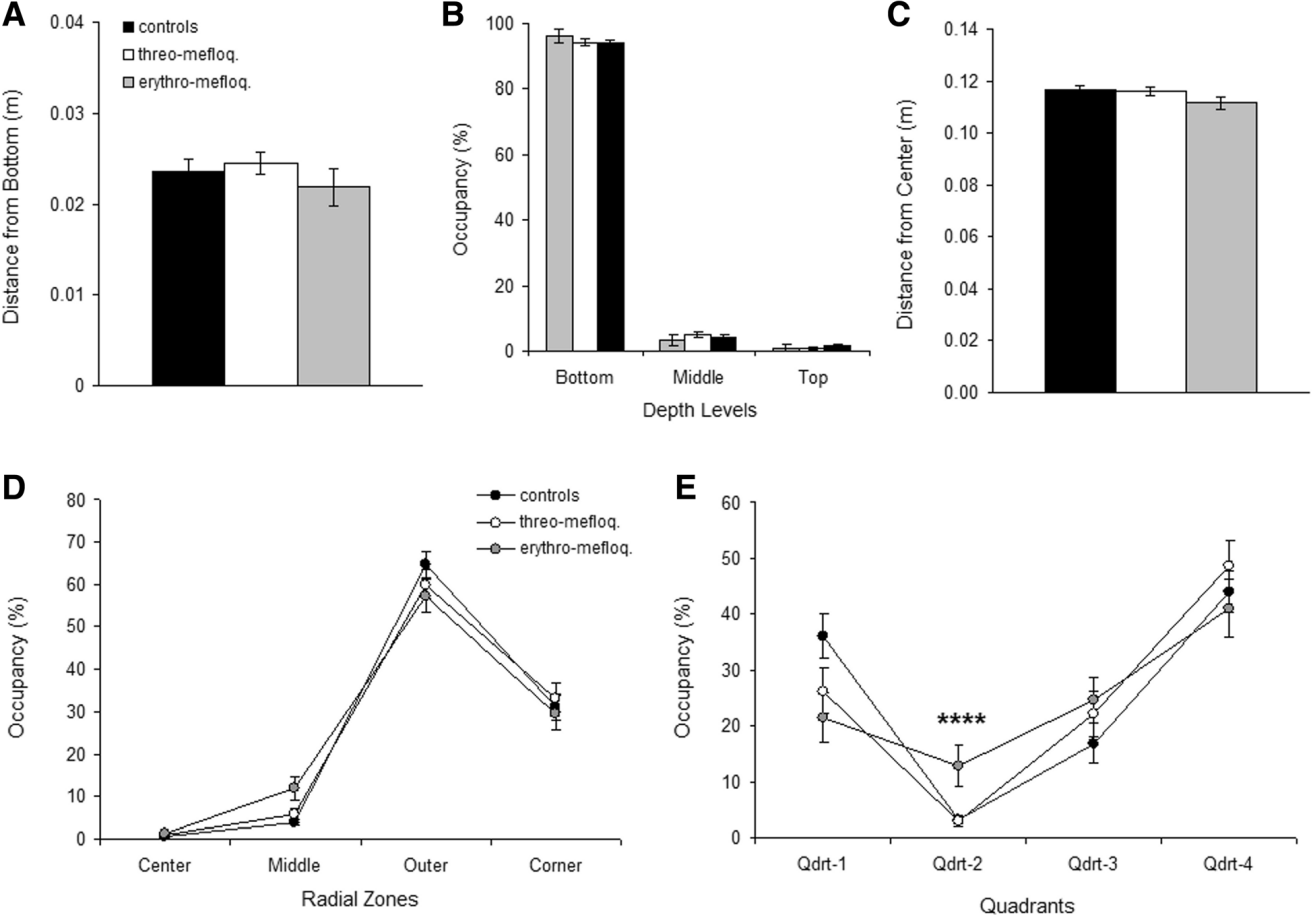
**Horizontal and vertical distribution. (A)** Average distance from bottom. **(B)** Relative time spent in bottom, middle and top third of observation container. **(C)** Average horizontal distance from center. **(D)** Relative time spent in any of the four radial zones. **(E)** Relative time spent in any of the quadrants. Mean ± SEM are presented. Comparison between the effects of erythro-mefloquine and water on time spent in quadrant 2: ****, p < 0.0001.

For average distance from bottom (Figure 4A), distribution over the three depth levels (Figure 4B), horizontal distance from center (Figure 4C) and distribution over radial zones (Figure 4D), no significant differences between treatments were observed. For distribution over the four quadrants (Figure 4E), Kruskal-Wallis test revealed an effect of treatment for quadrant 2 (H = 10.2, p < 0.01). The erythro-mefloquine groups spent significantly more time in that quadrant than the control group (p < 0.0001) and had a slight tendency to spend more time in it than the threo-mefloquine group (p = 0.021).

## Discussion

The preliminary experiment showed that during the 3 h of exposure to 43 mgL erythro-mefloquine all goldfish had died, but only 4.5% of the threo-mefloquine group perished. When lowering the dose to 21.5 mgL, 65% of the erythro-mefloquine group and 100% of the threo-mefloquine group survived. This was the first indication that threo-mefloquine had less toxic effects as compared to erythro-mefloquine, although a precise quantification was not performed. This latter was done by employing behavioral testing at a lower, non-lethal dose.

Behavioral testing of goldfish treated with a low dose (14.25 mgL) of either drug revealed that erythro-mefloquine significantly reduced 3D-travel distance and horizontal travel distance, whereas threo-mefloquine did not have such an effect. Duration freezing was also affected by the drug treatments; however, statistically this effect could not be further specified. Finally, both vertical and horizontal distributions were not affected by the drugs, except for the time the fish spent in quadrant 2. The goldfish treated with erythro-mefloquine spent more time in that quadrant. Not enough behavioral data are available for interpretation of our findings. Reduced locomotion and potentially increased duration freezing (Figure 3A, though not statistically demonstrated) could suggest sedation or increased anxiety in the erythro-mefloquine group. Furthermore, the preference for these fish for quadrant 2 could indicate increased anxiety, if we assume that goldfish behave similar as zebrafish in our testing apparatus [23].

Thus, both the preliminary experiments and the behavioral experiment suggest that in goldfish exposed to the drug dissolved in water, threo-mefloquine had less severe toxic effects as compared to erythro-mefloquine.

These finding seem to be consistent with *in-vitro* studies. For instance, Iglesias et al. [20] have demonstrated, that erythro-mefloquine (specifically Bioblocks QU024-1) reduced the magnitude of Pannexin-1 currents at a much lower dose than did threo-mefloquine (specifically Bioblocks QU025-1). Pannexin-1 is a trans-membrane protein that connects the intra- and extra-cellular spaces and that might play a role in, among others, neuronal processing [24]. Although the significance of this finding is not entirely clear, it might support the assumption that erythro-mefloquine has stronger pathological effects on neurological processes than threo-mefloquine. In another study, Gillespie et al. [25] have shown that the (−) enatiomer of erythro-mefloquine had a stronger affinity for the adenosine A_2A_ receptor than the (−) enantiomer of threo-mefloquine. The adverse neurological effects of mefloquine are according to Fletcher et al. [26] at least partly attributable to A_2A_ binding.

Interestingly, according to Karle et al. [27] in an *in-vitro* study threo-mefloquine was more effective than erythro-mefloquine against two clones of *Plasmodium falciparum*, D-6 and W-2.

However, according to Chaparro et al. [28], differences in pharmacokinetic properties explain why *in vivo* erythro-mefloquine is supposed to be more potent against malaria than threo-mefloquine. The confounding factor here is that they cite Basco et al. [29], who in turn cite the finding by Gimenez et al. [30] that (−)-mefloquine is present in a higher concentration in the plasma and whole blood compared to (+)-mefloquine after treatment with the drug. It should be pointed out that ‘mefloquine’ here means erythro-mefloquine (namely the R*S*-configuration, as described in [29]). To our knowledge no pharmacokinetic study comparing erythro- and threo-mefloquine has been performed in humans.

More important than pharmacodynamic and *in vitro* studies, are *in vivo* studies concerning the efficacy against malaria. According to Basco et al. [29] the findings of *in vivo* studies are inconsistent. For instance, in owl monkeys according to Schmidt et al. [31], erythro-mefloquine is about twice as effective as threo-mefloquine against *P. berghei*, but somewhat less effective than the latter against *P. falciparum* (which is the main target for mefloquine application). In mice, the experimental findings seem to be contradictory (according to [29]) in regard to its activity against *P. berghei*: one study [32] found that all four isomers were equally effective, whereas the other study (which was referred to, but not named in [29]) demonstrated that erythro-mefloquine was twice as effective as threo-mefloquine. This latter finding would be consistent with what Schmidt et al. [31] found for *P. berghei* in owl monkeys (see above). We conclude that the *in vivo* efficacies of erythro- and threo-mefloquine against malaria (especially *P. falciparum*) might need to be re-evaluated experimentally. Also, the pharmacokinetic properties of threo-mefloquine need to be investigated both in humans and in model animals.

## Conclusion

Taken together, our finding, that threo-mefloquine has less severe side effects than erythro-mefloquine in goldfish, is consistent with findings concerning the effect of both drugs on pannexin-1 currents and A_2A_ receptors. Moreover, the only published comparative *in vivo* study concerning the activity against *P. falciparum* we could find was done in owl monkeys and showed that threo-mefloquine was slightly more potent than erythro-mefloquine. In the current context it is interesting to note that small fish species might be an interesting model system for quick and relative low-cost testing of potential antimalaria drugs, at least in the early stages of drug development. For future studies, zebrafish might be preferred above goldfish because of the much shorter life cycle and the enormous knowledge gathered about this species in regard to genome and behavior. For basic toxicological testing, including organ toxicology, fish larvae might be in many cases a good model, especially since it offers the possibility of high-throughput screening. However, interference with ontogenetic aspects might be a limiting factor. Furthermore, adult fish allow a much broader assessment of the impact of drugs on behavior than is possible with larval fish. For further evaluating the therapeutic window of threo-mefloquine, testing for toxic effects after chronic drug application (in line with the prophylactic use of the drug) and applying more specific behavioral paradigms, such as reactivity to external stimulation (e.g. to test sensorimotor gating), neophobic and other anxiety-like responses, and social behavior (such as shoaling and mirror aggression) are future options to behaviorally characterizing neurobiological side effects of antimalarial drugs in this model system.

## Abbreviations

3D: Three-dimensional
A_2A_: A type of adenosine receptor
K: Kelvin (a measure of color temperature)
LED: Light-emitting diode
